# Alcohol, Drinking Pattern, and Chronic Disease

**DOI:** 10.3390/nu14091954

**Published:** 2022-05-07

**Authors:** María Barbería-Latasa, Alfredo Gea, Miguel A. Martínez-González

**Affiliations:** 1 Department of Preventive Medicine and Public Health, University of Navarra, 31008 Pamplona, Spain; mbarberia.3@unav.es (M.B.-L.); mamartinez@unav.es (M.A.M.-G.); 2 IdiSNA, Navarra Institute for Health Research, 31008 Pamplona, Spain; 3 Biomedical Research Network Center for Pathophysiology of Obesity and Nutrition (CIBEROBN), Carlos III Health Institute, 28029 Madrid, Spain

**Keywords:** alcohol, Mediterranean alcohol drinking pattern, Mediterranean diet, clinical trial, binge drinking, mendelian randomization, moderate consumption, abstinence

## Abstract

This review discusses the inconsistent recommendations on alcohol consumption and its association with chronic disease, highlighting the need for an evidence-based consensus. Alcohol is an addictive substance consumed worldwide, especially in European countries. Recommendations on alcohol consumption are controversial. On one hand, many nonrandomized studies defend that moderate consumption has a beneficial cardiovascular effect or a lower risk of all-cause mortality. On the other hand, alcohol is associated with an increased risk of cancer, neurological diseases, or injuries, among others. For years, efforts have been made to answer the question regarding the safe amount of alcohol intake, but controversies remain. Observational studies advocate moderate alcohol consumption following a Mediterranean pattern (red wine with meals avoiding binge drinking) as the best option for current drinkers. However, agencies such as the IARC recommend abstention from alcohol as it is a potent carcinogen. In this context, more randomized trial with larger sample size and hard clinical endpoints should be conducted to clarify the available evidence and provide clinicians with support for their clinical practice.

## 1. Introduction

Alcohol consumption represents an important global health problem and a priority for public health. The Sustainable Development Goals explicitly target the goal to strengthen the prevention and treatment of substance abuse including narcotic drug abuse and harmful use of alcohol [1]. Harmful alcohol use is ranked as the seventh global leading risk factor for death and disability [2]. Annually, three million deaths are attributed to alcohol, together with wider social harms that also extend beyond the drinker including intimate-partner violence (with a heavy burden on women), exacerbation of poverty, traffic injuries, and other effects [3].

Alcohol-related health problems are particularly severe in Europe. Europeans are the heaviest drinkers in the world, with an average intake of pure alcohol >25 g/d among adults, and a prevalence of current drinkers (in the last 12 months) of 72% (61.4% among women and 83.3% among men) [4,5]. The prevalence of heavy episodic drinking (≥60 g on ≥1 occasion during the past 30 days) was 30.4% in Europe. An estimate of 291,100 deaths in 2016 were attributed to alcohol consumption in Europe (5.5% of European deaths, 12% of premature mortality in men, and 2% in women) [5]. Alcohol represents the third leading factor for death and disability in Europe, responsible for €125 billion in costs per year [6].

The burden of disease associated with alcohol consumption is usually assumed to result from an imbalance between beneficial and harmful effects. Nonrandomized epidemiologic studies have attributed some benefits to moderate alcohol consumption on ischemic heart disease, diabetes, or ischemic stroke, whereas they found detrimental effects on injury, suicide, several types of cancer, liver disease, mental disorders, and communicable diseases [2]. Considering the net balance, simple and generalized messages have been issued such as the safe level of alcohol intake should be zero [3,7,8,9,10,11] and “no level of alcohol consumption improves health” [7].

Absolute average alcohol intake does not seem to fully explain the shape of the dose–response relationship observed between alcohol intake and cardiovascular disease (CVD). Some aspects of the drinking pattern might act as effect modifiers including heavy episodic intake (binge drinking), beverage preference, consumption with or without meals, and distribution throughout the week [12,13,14,15,16,17,18,19,20,21]. Large cohorts concluded that ‘healthy’ patterns of moderate drinking reduce CVD [12,13,22,23,24,25,26,27,28,29,30], diabetes [31], and even all-cause mortality [32,33,34,35,36,37,38], particularly in subjects ≥50 years [26,29,39,40]. However, these cohorts included highly selected middle-to-high class subjects, highly educated subjects, or only health professionals. They also reported J-shaped associations for all-cause mortality. These effects might be dependent on the distribution of causes of death in the selected samples and on the distribution of levels of alcohol consumption in cohort participants, with few of them drinking high amounts or indulging in binge drinking [11]. This fact may compromise the strength of their potential causal inferences and generalizability [41,42,43,44,45,46].

In addition, studies using genetic instrumental variables or Mendelian randomization (MR) analyses have challenged that moderate alcohol consumption may reduce CVD or total mortality and support complete abstention as the healthiest option [47,48,49,50]. These analyses avoid the problems of self-selection for the drinking pattern, thus reducing confounding. They also provide a proxy of lifetime consumption and eliminate reverse causation. There are also limitations in MR analyses because, while MR analyses may contribute to identifying causal pathways, they cannot quantify the dose–response relationship between levels of alcohol consumption and mortality [51]. Several limitations specific to the genetic underpinnings of Mendelian randomization analyses do not allow them to be fully equiparated to randomized trials [51,52,53]. Some assumptions of the MR analyses have been challenged when analogies for achieving causal inferences have been made between the effect of the genetic variant in a MR study and the intention-to-treat effect in a randomized controlled trial (RCT).

There are four weaknesses in such an analogy, as reported elsewhere in further detail [53]. First, the compared groups with different genetic variants in MR may not be balanced, as they are in large RCTs, because of linkage disequilibrium, population stratification, or other reasons. Second, the genetic variant is not always causal, but it might only be a misclassified version of an assigned treatment strategy and the important assumption of a strong homogeneity effect within each group required for the use of instrumental variables is usually not met in MR studies because it would be biologically implausible. Third, the definition of treatment “adherence” (that permits one to differentiate between intention-to-treat and per-protocol analyses) is unclear in MR and cannot be properly defined because the treatment strategies are not explicitly described. Fourth, and very importantly, “time zero” of follow-up (i.e., the inception instant in a trial) is usually not well defined in a MR analyses. In a RCT, “time zero” corresponds to the coincidence of four facts: the moment when the eligibility criteria are met, random allocation, the onset of the treatment strategy, and the starting time for counting the events of interest (outcomes). This does not occur in MR studies where a lag of several decades exists between the time of pseudo-randomization (“treatment”), the time of eligibility, and the onset of outcome recordings. These timing mismatches do not sustain a perfect analogy between a MR analyses and a RCT for causal inference. Thus, MR studies, despite contributing to identifying causal pathways, cannot replace large and well conducted RCTs [52,53].

Modeling studies such as the Global Burden of Disease (GDB) [2,4,5,6,54] are also currently invoked to support that total abstention is the healthiest alcohol dose [3,7,8]. Despite their utmost importance, these studies are in part based on some unfounded postulates [51]. They assume that true causal measures of effect are known for all relevant diseases and in all continents and countries as well as in the homeless, the less well-off strata, and other sectors of the population. They also assume that all relevant effect modifiers have been identified (to ensure extrapolation of previous estimates obtained in highly selected cohorts to external populations) and that accurate prevalence estimates are available for all countries. However, this is not the case [41,51,54,55,56]. These models are likely to be highly sensitive to the selection and precision of their inputs, which are not always explicitly disclosed. For example, tuberculosis importantly contributed to the estimated disease burden attributed to alcohol [2], but the GBD study included tuberculosis and not Hodgkin’s disease or other hematologic malignancies that previously showed inverse associations with alcohol [54,57]. They also ignored the effect of the drinking pattern (problematic drinking is considerably more important for tuberculosis than moderate alcohol intake) [58,59]. Additionally, an increased risk of colorectal cancer is only supported for >3 drinks/d [60], however, a purely linear dose–response shape was assumed by the GBD.

Several mechanistic studies have attempted to explain the pathways that may link alcohol intake to the pathogenesis of diseases. Both cell damage and protective mechanisms have been found depending on the dose of ethanol. High concentrations of alcohol trigger oxidative and cardiotoxic mechanisms in cells causing cardiomyopathy, arrhythmias, and heart failure [61,62]. Underlying mechanisms that associate alcohol consumption with the development of diabetes mellitus in mice have also been found [63]. On the other hand, low and even moderate concentrations of alcohol caused anti-fibrillatory effects in atria [61] as well as increased HDL cholesterol levels and improved heart energy metabolism profile [64]. Resveratrol has also been proposed as an anti-fibrillatory agent through the modulation of ROS and oxidative stress [65]. Moreover, the mechanisms involved in the association between alcohol and cancer have also been extensively studied, especially for breast cancer, where they highlighted the harmful effects of ethanol on breast cancer cells such as growth promotion and angiogenesis [66,67]. They also found that cells repeatedly treated with alcohol increased their ability to migrate and metastasize other tissues [67,68,69,70]. Despite the limitations presented by the in vitro and in vivo studies, they suggest possible mechanisms underlying the association between alcohol intake and the risk of chronic disease.

Alcohol was classified as a group 1 carcinogen 30 years ago, and it imposes a severe toll on cancer, being responsible for 4.1% of all new cases of cancers in 2020 [6]. Alcohol is causally linked to cancers of the oral cavity, pharynx, larynx, liver, esophagus (squamous cell carcinoma), breast, and colo-rectum [6,59,60,71]. Even moderate levels of consumption (about 1–2 drinks/d) have been found associated with higher risks including cancer of the breast [71]. Nevertheless, some important cohorts have reported inverse associations of moderate alcohol intake with total cancer. The European Prospective Investigation into Cancer and Nutrition (EPIC) found the lowest cancer risks for alcohol intake between 6 and 25 g/d. Even for ‘alcohol-related’ cancers, the lowest risk was found for 12–25 g/d [72]. Two large American cohorts reported the lowest risk of cancer death for participants with consumptions of 1–5 g/d, not for abstainers [37,73].

The drinking pattern may act as an effect modifier even for the relationship between alcohol intake and liver cirrhosis. In fact, a recent analysis found a 31% relative reduction in the risk of cirrhosis among drinkers with meals compared to drinkers outside meals, adjusting for the quantity consumed [74,75,76].

Specifically, wine consumption, particularly in Mediterranean countries, has been postulated as a key feature of the Mediterranean diet with strong cardio-protective properties [77,78] due to the abundance of polyphenols in red wine with postulated antioxidant or anti-inflammatory effects [79,80,81,82]. Our group also found an interaction between alcohol intake and the overall drinking pattern on mortality [14,16,19], concluding that a traditional Mediterranean alcohol drinking pattern (MADP) (i.e., a moderate consumption of red wine during meals, spread out throughout the week, and avoidance of binge-drinking) was associated with lower mortality compared to abstention or the departure from this MADP within constant levels of alcohol intake. A modification of the effect of the amount of alcohol intake on total mortality by the drinking patterns has been subsequently confirmed by recent large prospective studies conducted in the UK Biobank [75,76].

## 2. Methodological Problems in Nonrandomized Studies

The “sick quitter” effect and the “healthy user” effect are well-known methodological concerns of observational (i.e., nonrandomized) studies on alcohol. The first effect is explained because the group of non-drinkers may include former drinkers who quit due to illness, and the second one because healthier subjects are more likely to self-select themselves to drink moderately and more responsibly; this may confound the association between moderate alcohol intake and clinical outcomes and might provide a likely non-causal explanation on why, in some nonrandomized studies, abstainers showed a higher risk of chronic disease or all-cause mortality than moderate consumers of alcohol [83]. In addition, residual confounding by socioeconomic status, social integration, diet, lifestyles, health consciousness, and self-control cannot be ruled out in nonrandomized studies. In fact, in some meta-analyses, careful adjustment for potential confounders and other potential non-causal factors attenuated the reduction in mortality observed in moderate consumers [83,84,85]. A different caveat is that self-reported alcohol intake may lead to misclassification (underestimation of the true amount consumed in heavy drinkers) due to social desirability or unacceptability biases. Heavy drinkers may tend to underreport their true levels of alcohol consumption by up to 40% to 65% [86,87,88,89]. Systematic underreporting of alcohol use by heavy drinkers could result in overestimating the risks of adverse health outcomes associated with self-reported low-to-moderate amounts of alcohol, finding a non-true linear dose-dependent relationship with these adverse health outcomes, given that some heavy drinkers might be misclassified as only moderate drinkers due to their underreporting [87,89].

These speculations on nonrandomized studies are difficult to test or, in some cases, they have even been contradicted by empirical findings [40,79,80,81,82]. In fact, a large study reported that patients with certain medical conditions actually maintained higher alcohol intake than healthy subjects, thus contradicting the “healthy user” effect [90]. However, other data do support the “sick-quitter” and “healthy user” biases [91].

## 3. Diverging Recommendations

**Figure 1:**
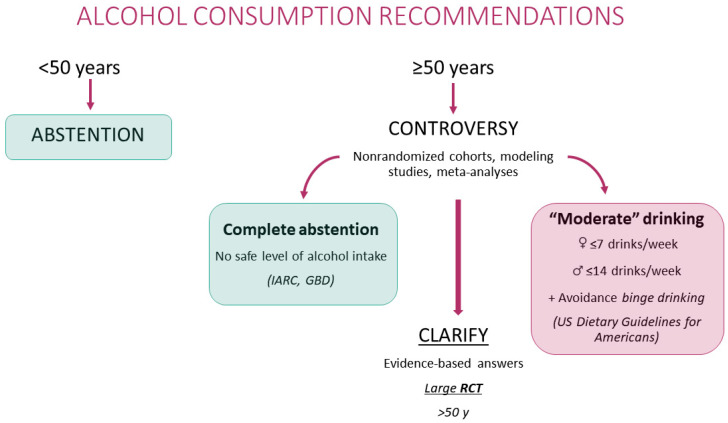
Diverging recommendations on alcohol consumption.

In this complex situation, a controversy currently exists regarding which is the best and most practical and realistic advice for drinkers aged 50–70 years old (Figure 1). Two completely opposite points of view are defended. On one hand, one point of view holds that complete abstention is the healthiest option for them, affirming that “there is no safe level of alcohol intake” for anybody and implying a strong need to reduce average alcohol consumption in all sectors of the population regardless of their initial consumption levels, their age, and their baseline risk. On the other hand, a harm reduction strategy for these adults leading to recommending “moderate” drinking (≤7 drinks/week in females and ≤14 in males) and avoidance of binge drinking is supported by wide sectors of the public health community including the U.S. Dietary Guidelines for Americans (DGA) 2015–2020, which recommended ≤2 drinks/d for males and ≤1 for females in 2015. The scientific report for the U.S. DGA 2020–2025 recommended moderation with ≤1 drink/d for both males and females, without substantial new evidence. Interestingly, in large U.S. cohorts, intakes of 5–15 g/d for women and 5–30 g/d for men represented one of the five elements of a lifestyle score robustly associated with longer life expectancy [37].

Therefore, both contradictory advices are apparently supported by nonrandomized cohorts, modeling studies, and meta-analyses [10,20,26,33,39,40,41,42,43,44,46,47,48,55,80,84,85,92]. This paradoxical situation is usually addressed only with plausible assumptions and speculations, but not with the needed firm evidence. Clinicians need evidence-based answers to clarify this controversy. Before opting for any of the two alternatives, the most sensible approach to overcome these controversies and the inherent limitations of measurement errors, selection biases, reverse causation, residual confounding, and misclassification would be to conduct a large RCT of alcohol with hard clinical endpoints among subjects older than 50 years. This task would represent a groundbreaking investigation with high risks and high gains, going beyond the current state-of-the-art. It would require interdisciplinary work and could contribute enormously to the advancement of science by gaining sound evidence-based responses to the relevant and long-pending issue of what is the ideal option for alcohol intake among adults 50–70 years.

This large and groundbreaking RCT is long overdue and has largely been warranted for decades. Given the potential biases inherent to nonrandomized studies [93,94,95], they may not be valid when the expected effects are likely to be actually null or only moderate, with relative risks less than two-fold [95]. This is most probably the case for the overall effects of light to moderate alcohol intake. As recently suggested by Collins et al., “large observational studies may yield misleading associations of a treatment with health outcomes that are statistically significant but non-causal, or that are mistakenly null when the treatment really does have clinically important effects. Instead, randomized, controlled trials of adequate size are generally required to ensure that any moderate benefits or moderate harms of a treatment are assessed reliably enough to guide patient care appropriately” [95].

In the era of evidence based-medicine and given the abundance of alcohol intake worldwide, it is surprising that no large RCT has ever been conducted to appropriately test the effects of alcohol. A previous attempt, the MACH15 trial, although nicely-designed and thoroughly planned, was terminated by the NIH and the funding withdrawn because of some suspicion on issues related to potential conflicts of interest with the alcohol industry. These issues were not clear. However, this unprecedented study was started and demonstrated the feasibility and timeliness of a trial on alcohol intake [96,97]. Unless a large RCT is conducted, discussions on alcohol issues upon mere assumptions and speculations on nonrandomized studies seem useless. A clear and definitive answer based on first-level evidence is of utmost need. A RCT should assess the effects of moderation versus abstention/reduction of alcohol intake on a holistic and comprehensive composite outcome including cardiovascular disease, cancer, other chronic diseases, injuries, major infectious diseases, and all-cause mortality, as previously claimed [55,56]. Although it may appear heterogeneous such a composite end-point is needed because alcohol is related to many (>200) individual diseases [4,9,41]. The balance between potential benefits and harms needs to be adequately considered if a large RCT is going to be started. Unless a large RCT is completed, starting from an agnostic perspective and free of competing interests, the needed first-level scientific evidence will continue elusive, and futile debate will continue for decades to come.

Funding agencies in the 21st century might risk leaving future generations without the needed knowledge and insights into the health effects of one of the most widely used substances by humanity for millennia, with Europeans currently as the highest consumers. The answer to this question also includes important economic and business consequences. Potential conflicts of interest highlight the need to publicly fund this RCT. Public funding and avoidance of any funding provided by the alcohol industry is key for an a priori completely agnostic approach, with no preferred expectations on any of the two compared alternatives, namely, abstention versus moderation.

## 4. Existing Randomized Controlled Trials in the Field of Alcohol Intervention

A number of short-term randomized feeding studies, conducted in small numbers of participants, have tested the effects of alcohol on several intermediate mechanisms. They predominantly examined the effects on lipids, inflammation, and glycemia [98], but not on hard clinical endpoints and cannot replace a formal large RCT. Alcohol intake raised the levels of high-density lipoprotein cholesterol (HDL-C) and apolipoprotein A1. Additionally, alcohol intake increased adiponectin, an adipokine that increases insulin sensitivity and is inversely associated with diabetes risk [98], reduced fasting levels of insulin and glycosylated hemoglobin [99], circulating levels of fibrinogen and prolonged bleeding time, thus affecting platelet function [100]. Other mechanistic findings are related to the effects of alcohol in increasing sex steroid hormones, particularly estrone [101] and dehydroepiandrosterone sulfate [102]. Alcohol exposure (three to four months in women) found important hormonal effects [102,103]. In the EPIC cohort, a formal mediation analysis reported that an alcohol-related hormonal signature (negatively associated with sex-hormone binding globulin and positively with estradiol and testosterone) was associated with increased breast cancer risk [104].

The longest trial to date of alcohol was the CArdiovaSCulAr Diabetes and Ethanol (CASCADE) trial, which lasted two years and included 224 participants with well-controlled type 2 diabetes [105]. All participants were initially alcohol abstainers, and they were randomly assigned 1:1:1 to consume, with dinner, 150 mL of mineral water, white wine, or red wine. Subjects randomized to initiate red wine increased their HDL-C and Apo A1 and reduced their number of components of the metabolic syndrome as compared to the mineral water group. No differences were found in other outcomes (glycemic control, blood pressure, adiposity, liver function, or quality of life). In Italy, a RCT with 131 patients with myocardial infarction and diabetes (Mediterranean-type diet with or without the addition of four daily ounces of red wine in a 1:1 ratio) reported higher levels of HDL for red wine, lower levels of oxidation markers, reductions in several inflammatory biomarkers, lower fasting insulin levels, and improved left ventricular function after one year [106]. Other small RCTs also tested the intermediate effects of alcohol including counseling to reduce intake [107,108,109,110,111,112,113,114,115,116,117,118,119].

Recently, a 6-month RCT in Australia with 140 drinkers (consuming approximately 120 g/week of pure alcohol at baseline) concluded that abstinence from alcohol reduced atrial fibrillation (AF) recurrences among drinkers with previous AF, who were in sinus rhythm at baseline [120]. The steering committee revised the protocol and shortened the follow-up to six months, instead of one year, because they did not find enough participants willing to abstain from alcohol for 12 months. This was the first RCT of alcohol with a clinical endpoint, and patients in the ‘abstinence’ group reduced their alcohol intake from 16.8 ± 7.7 to 2.1 ± 3.7 drinks/week (an 87.5% reduction), with complete abstinence achieved by 61% of participants in the abstinence group. Surprisingly, apart from these 140 drinkers to date, there is no other RCT in a free-living population evaluating the risk of any other major clinical outcome for two alternative advices on alcohol.

## 5. Mediterranean Alcohol Drinking Pattern, Wine in Moderation, and Reduced All-Cause Mortality

Previously, we operationally assessed the Mediterranean alcohol drinking pattern (MADP), defined as a moderate consumption of red wine during meals throughout the full week and avoidance of binge-drinking [14]. This MADP was inversely associated with all-cause mortality compared to alcohol abstinence. This is consistent with the so-called “French paradox”, a term coined three decades ago to try to explain the low rates of heart disease in the French population, together with a diet rich in saturated fats [121,122,123,124]. This paradox was attributed to usual wine consumption with meals [78,79,80,81,121,122,123,124,125,126,127,128,129,130]. The Greek EPIC cohort reported that none of the other nine items used to define the Mediterranean diet exerted such a strong benefit as moderate alcohol intake did on all-cause mortality. In that cohort, alcohol intake mainly came from wine consumed during meals [131]. These findings support the basis to defend the modifying effect of wine preference (as part of the drinking pattern) on the effect of total alcohol intake on all-cause mortality. Given the addictive nature of alcohol, and the inherent practical difficulties to achieve and maintain abstention (or to get substantial reductions) in drinkers, a harm-reduction approach (namely, the MADP) can be appropriate for drinkers insofar as it is supported by scientific evidence.

## 6. Mediterranean Diet and Mediterranean-Alcohol Drinking Pattern, A Complex Relationship

Moderate alcohol consumption is one of the main components of the Mediterranean diet, which greatly contributes to the reduction in total mortality risk [131,132]. Many studies have focused on the determination of a safe level of alcohol intake, with mixed results from abstinence to moderate consumption [10,20,26,40,41,42,43,44,55,84,85]. However, rather than looking at each individual component, more attention should be paid to the overall pattern of consumption [78,133]. A large number of prospective studies reported a greater benefit from adherence to a Mediterranean dietary pattern than from the intake of a particular nutrient [133,134,135,136]. Therefore, an a priori Mediterranean pattern of alcohol consumption was proposed with positive results on mortality in Mediterranean cohorts [14,16,17,19,21,131,137]. These results suggest the existence of multiple coexisting and interrelated mechanisms. Antioxidant components of the diet and wine may reduce the carcinogenic effect of ethanol [138]. However, the evidence is limited and suggests a lower risk of all-cause mortality with high adherence to the Mediterranean dietary pattern together with MADP [19]. This complex relationship between diet and alcohol requires new lines of research to give more precise recommendations. In the absence of a RCT providing a higher level of evidence on alcohol consumption patterns, abstainers should not be recommended to start drinking, but those who do drink should be encouraged to follow the MADP in combination with the Mediterranean dietary pattern [19]. Once again, there is a clear need for an experimental trial to clarify the relationship between diet and alcohol to answer the question of a safe level of consumption.

## 7. Alcohol Consumption and Chronic Diseases

Alcohol consumption is associated with a higher risk of acute diseases (infections, injuries, traffic accidents, violence, fetal alcohol disorders, among others) and variable effects on chronic diseases (diabetes mellitus, digestive diseases, cancers, CVD). The harmful effects of alcohol are largely influenced by the total amount of ethanol ingested, the drinking pattern, and the specific type of alcoholic beverage consumed [12,13,14,15,16,17,18,20,21]. This is even more important when it comes to chronic alcohol consumption (Table 1). Several studies have associated repeated exposures to large amounts of ethanol in the blood with increased oxidative stress. This effect is likely to occur because during alcohol metabolism, reactive oxygen species (ROS) and nitrogen species (RNS) are produced, which cause oxidative stress, cell damage, inflammation, and DNA oxidation, leading to mutations. It also has the potential to decrease antioxidant activity and can lead to chronic diseases such as diabetes, organ inflammation, cardiovascular problems, or cancer [139,140,141].

### 7.1. Alcohol and CVD

Several nonrandomized studies have defended a J-shaped curve for the association between alcohol consumption and cardiovascular disease, with protective effects attributed to moderate consumption [12,13,16,17,22,23,24,25,26,27,28,29,30]. They emphasized the modifying effect of a moderate or healthier drinking pattern, particularly in subjects over 50 years of age [12,13,14,15,16,17,18,19,20,21,26,29,39,40], on potential alcohol beneficial effects on blood pressure, atrial fibrillation, or stroke [13,16,17,156]. One explanation for these potential benefits may lie in the antioxidant properties of wine, although there is still a large research gap to be filled [80,81,82,83]. On the other hand, some studies have argued for a linear association of alcohol with CVD or mortality [142,143,144] and attributed methodological problems and biases to the effects of moderate consumption and advocated zero alcohol [47,48,49,50].

### 7.2. Alcohol and Diabetes

Low-moderate alcohol consumption has been associated with reduced insulin resistance and HbA1c levels in non-diabetic patients and lower cardiovascular risk in diabetics [31,99,145]. However, there are studies that have challenged this claim as their findings also showed that diabetic patients who consumed alcohol adhered less to their treatment [146,147]. However, it is true that they agreed that chronic heavy drinking causes a disruption in glucose homeostasis, increases insulin resistance, and thus the risk of diabetes [99,146].

### 7.3. Alcohol and Digestive Diseases

The harmful effect of alcohol on the liver is well-known, leading to diseases such as hepatitis, chronic liver disease, and cirrhosis. Ethanol is metabolized in the liver, generating oxidative compounds and the accumulation of fatty acids. Therefore, hepatitis C or alcoholic fatty liver disease are the result of chronic high alcohol consumption [2,4,149,150,151,152,153]. Furthermore, although the liver is the main organ affected, high alcohol consumption can lead to pancreatitis [149,154,155] or gastritis [153]. However, it is important to note that moderate patterns do not seem to be as associated with these diseases as high consumption [148].

### 7.4. Alcohol and Cancer

Cancer, like cardiovascular disease, is one of the leading causes of death in the world. Due to the oxidative compounds caused by alcohol metabolism, cells are more prone to mutations and therefore a direct link to cancer is assumed. Most organizations advocate zero alcohol consumption to prevent breast, oropharynx, larynx, larynx, esophagus, liver, colon, and rectum cancers [2,4,6,59,60,71,72]. However, similarly to previous diseases, cohort studies have found that low or moderate alcohol consumption is associated with a lower risk of cancer than the abstainer group [37,73,74].

## 8. Conclusions

Despite the possible limitations in the available evidence summarized in this review, the controversy regarding the potential effects of light to moderate alcohol consumption in subjects older than 45–50 years remains open. Clinicians need a sound, evidence-based, answer to this question to provide recommendations to their patients. Unless a long-term randomized clinical trial with hard clinical events as the end-point is conducted, this controversy will remain, and regardless of how large the cohort studies or meta-analyses of the nonrandomized study may be, the definitive answer will remain elusive.

## Figures and Tables

**Table 1:** Alcohol consumption and chronic diseases.

Protective Effects	Negative Effects
Alcohol and CVD	
Drinking pattern: low-moderate alcohol (♀ ≤7drinks/week, ♂ ≤14 drinks/week) + with meals + avoid binge drinking	Chronic alcohol consumption, heavy drinking
Effects on blood pressure, atrial fibrillation, or strokes	High blood pressure, cardiomyopathy, coronary heart disease
Articles: [12,13,16,17,21,22,23,24,25,26,27,28,29,30,39,40,141]	Articles: [47,48,49,50,142,143,144]
Alcohol and Diabetes Mellitus	
Low-moderate alcohol consumption	Chronic alcohol consumption, heavy drinking
Reduced insulin resistance, HbA1c levels and CVD risk	Disruption in glucose homeostasis, higher insulin resistance, less adherence to diabetes treatment
Articles: [31,99,145]	Articles: [99,146,147]
Alcohol and Digestive diseases	
Low-moderate consumption	Chronic alcohol consumption, heavy drinking
-	Hepatitis, chronic liver disease, cirrhosis, pancreatitis, gastritis
Articles: [148]	Articles: [2,4,149,150,151,152,153,154,155]
Alcohol and Cancer	
Low (1–5 g/day), moderate (12–25 g/day)	All alcohol consumption, especially heavy and chronic patterns
Lowest risk of cancer (even alcohol related cancers)	Higher risk of breast, oropharynx, larynx, larynx, esophagus, liver, colon and rectum cancers
Articles: [37,73,74]	Articles: [2,4,6,59,60,71,72]

## Data Availability

Not applicable.
